# 

**Published:** 1886-11

**Authors:** 


### SPECIAL.

We desire to inform our readers that, commencing with the coming year, the JOURNAL will be published punctually on the first of every month instead of the middle of the month as heretofore.

We would also call attention to the list of premiums to subscribers advertised in our October number. The offer is still open for all who desire to avail themselves of it.

The volume commencing January 1st will be the thirty-fourth. This journal has never been published under any other name than that of "HALL'S JOURNAL OF HEALTH." We have no connection with any other publication, nor have we any proprietary interest in any medicinal remedy advertised in its columns. We make this statement as a final answer to correspondents asking information on this subject. It will always be our policy to occupy a neutral position in respect to our advertising patrons, who may rest assured that we will never present a medicine of our own in competition with theirs, nor shall we permit ourselves to advertise as beneficial, any lotion or compound, unless satisfied that it is substantially what it claims to be.

### DREAMS.

(*Continued.*)

### BOOK NOTICES.

The following publications have been laid upon our table since issuing the October number :

"A Manual of Animal Vaccination," by Dr. E. Warlomont, member of the Royal Academy of Medicine, of Belgium, etc. Translated by Arthur J. Harris, M. D. Published by John Wyeth & Bro., Philadelphia.

The author of this practical treatise was a leader in the movement to establish animal vaccination as a preventive of small pox, and founder of the vaccine bureau of the Belgian Government, and for a long period its official head ; and its translator is Consulting Physician to the Association for the supply of pure vaccine lymph, of Philadelphia, hence we may take it for granted that this manual contains the fullest obtainable information upon the subject of which it treats. In looking over its pages we are fully borne out in this assertion. In addition to this, the treatise contains several illustrations of instruments and methods of inoculation with men and animals. It will doubtless be found of great value to the profession.

"Manual of Psychometry : The Dawn of a New Civilization." By Joseph Rodes Buchanan, M.D. 2d edition, published by the author.

Professor Buchanan is too well known as one of the most distinguished among scientists of all nations to require any introduction to the public at our hands. The book in question is the latest of his published works, and contains, among other valuable information, the details of a large number of psychrometric readings by Mrs. Buchanan (formerly Mrs. Decker), for which she has long been noted. For any further informa- on the subject, the reader is referred to the article on the author of this book, in another part of the present issue.

The October "Century," in addition to its interesting war and miscellaneous articles, contains the first chapters of a biography of Abraham Lincoln, by J. G. Nicolay and John Hay, who from having served that illustrious statesman and martyr to liberty in the capacity of Private Secietaries, during the troublous days of the great rebellion, must be admirably equipped for this important undertaking which is to be continued in forthcoming numbers.

